# HPV testing with 16/18 genotyping for risk stratification among women with normal cytology: a multicenter prospective cohort study from China

**DOI:** 10.1128/jcm.01289-25

**Published:** 2026-02-19

**Authors:** Jiangong Zhang, Hong Wang, Yin Liu, Zhifang Li, Xiangxian Feng, Xiping Luo, Wen Chen, Shaokai Zhang, Hui Yang, Youlin Qiao

**Affiliations:** 1 School of Life Sciences, Northwestern Polytechnical University 26487, Xi'an, China; 2 Department of Cancer Epidemiology and Prevention, Affiliated Cancer Hospital of Zhengzhou University/Henan Cancer Hospital, Henan Engineering Research Center of Cancer Prevention and Control, Henan International Joint Laboratory of Cancer Prevention, Zhengzhou, China; 3 Department of Preventive Medicine, Changzhi Medical College 74652, Changzhi, China; 4 Department of Gynecology, Guangdong Women and Children Hospital 90405 , Guangzhou, China; 5 Department of Epidemiology, National Cancer Center/National Clinical Research Center for Cancer/Cancer Hospital, Chinese Academy of Medical Sciences and Peking Union Medical College 12501, Beijing, Beijing, China; 6 School of Population Medicine and Public Health, Chinese Academy of Medical Sciences & Peking Union Medical College 569801, Beijing, China; Wadsworth Center - NYSDOH, Albany, New York, USA

**Keywords:** cervical cancer, high-risk human papillomavirus testing, genotype, screening, cervical intraepithelial neoplasia

## Abstract

To evaluate the clinical performance of Hybribio’s 14-type HPV real-time PCR with 16/18 genotyping (HBRT-H14) and its risk stratification utility among women with normal cytology (NILM). From 2017 to 2020, a multicenter cohort enrolled 8,401 women aged 30–64 years with NILM cytology. Baseline HPV testing used HBRT-H14. Women positive for HPV 16/18 were referred for colposcopy; follow-up was annual for 3 years or until the detection of cervical intraepithelial neoplasia grade 2 or worse (CIN2+). Analyses included 6,679 women who completed follow-up. Overall HPV positivity was 11.4%, including 2.3% HPV 16/18. Over 3 years, sensitivity and specificity of HPV positivity for CIN2+ were 92.3% (95% confidence interval [CI]: 84.2–96.4) and 89.6% (88.8–90.3). For HPV 16/18 positivity, sensitivity and specificity were 41.0% (30.8–52.1) and 98.2% (97.8–98.5). Three-year cumulative CIN2+ risk was 20.9% (15.2–28.1) for HPV 16/18-positive women, 6.6% (4.9–8.9) for other types, and 0.1% (0.04–0.2) for HPV-negative women. HBRT-H14 shows strong clinical performance for detecting CIN2+, and HPV 16/18 genotyping provides effective risk stratification among women with NILM cytology. Findings support integration of HBRT-H14 into HPV-based screening pathways with HPV 16/18 genotyping and cytology triage of other types.

**IMPORTANCE:**

This multicenter prospective study evaluated the Hybribio 14 high-risk HPV real-time PCR assay (HBRT-H14) in 8,401 women with normal (NILM) cytology under guideline-based follow-up. The assay showed high clinical sensitivity and a very low risk among HPV-negative women, and HPV 16/18 genotyping provided clear risk stratification. These findings deliver large-scale, practice-oriented evidence supporting integration of HBRT-H14 into HPV-based screening pathways that use HPV 16/18 genotyping with cytology triage of other types.

## INTRODUCTION

Cervical cancer remains a major global health burden, with an estimated 660,000 new cases and 350,000 deaths in 2022 (1). China accounts for more than one-fifth of all cases, and incidence has continued to rise despite organized efforts (2), underscoring the need for more effective screening strategies.

Because most cervical cancers arise from persistent infection with HPV, particularly types 16 and 18 (3), HPV testing has emerged as a more sensitive approach than cytology for detecting precancerous lesions. Evidence from large randomized trials has demonstrated that HPV-based screening provides superior sensitivity and long-term prevention compared with cytology (4–8). Moreover, primary HPV testing with cytology triage has been shown to be more efficient and cost-effective than cotesting (9, 10). Consequently, international guidelines increasingly recommend primary HPV testing as the preferred screening approach (11), while cotesting or cytology alone remains acceptable alternatives in settings where primary HPV testing has not yet been fully implemented (12–14).

Multiple HPV assays are commercially available, but only a subset meet clinical validation criteria for use in screening (15, 16). The Hybribio’s 14 HR-HPV with 16/18 genotyping real-time polymerase chain reaction (PCR) assay (HBRT-H14) targets 14 HPV types (16, 18, 31, 33, 35, 39, 45, 51, 52, 56, 58, 59, 66, and 68), with specific genotyping for HPV 16 and 18. While its analytical accuracy has been validated (17), its clinical performance and risk stratification utility within screening pathways require prospective evaluation.

Given that screening strategies and readiness for HPV-based programs differ across countries, assessing clinically validated assays within local practice settings is critical to support evidence-based policy adoption. Although many countries have transitioned to primary HPV screening, cotesting remains widely implemented in China during this transitional period. Therefore, this study aimed to evaluate the risk-stratification performance of the HBRT-H14 assay in a national multicenter prospective cohort of women with NILM cytology, in accordance with Chinese guidelines (18). The findings are expected to provide crucial evidence supporting the integration of HBRT-H14 into HPV-based cervical cancer screening pathway through HPV 16/18 genotyping and cytology triage of other high-risk types.

## MATERIALS AND METHODS

### Study design and participants

This multicenter prospective observational study was conducted in China from April 2017 to December 2020. Eligible women were recruited from cervical cancer screenings in Henan, Shanxi, and Guangdong Provinces. Women were included if they (1) were aged 30-64 years with an intact cervix; (ii) maintained good health and compliance with cervical cancer screening protocols (colposcopy, biopsy); (iii) signed an informed consent form; and (iv) presented with a NILM cytology result. Women were excluded if they (i) were pregnant or within 8 weeks postpartum; (ii) had a history of cervical surgery or pelvic radiotherapy; or (iii) had a history of cervical cancer or precancerous lesions.

### Sample size calculation

On the basis of the prospective study data, the cumulative incidence rate of cervical intraepithelial neoplasia grade two or worse (CIN2+) over 3 years in the baseline population with NILM cytology ranged from 1.1% to 2.1% (19). According to guidelines for the technical review of HPV nucleic acid detection and genotyping reagents in China, the number of cases with CIN2+ at the end of follow-up should be no less than 60 (18). Therefore, it is estimated that the sample size of the population with NILM is 60/1.1% = 5,310 cases. Considering a 30% missed follow-up rate, approximately 7,586 women with NILM were planned to be included in this study.

### Baseline screening and follow-up procedures

**Fig 1:**
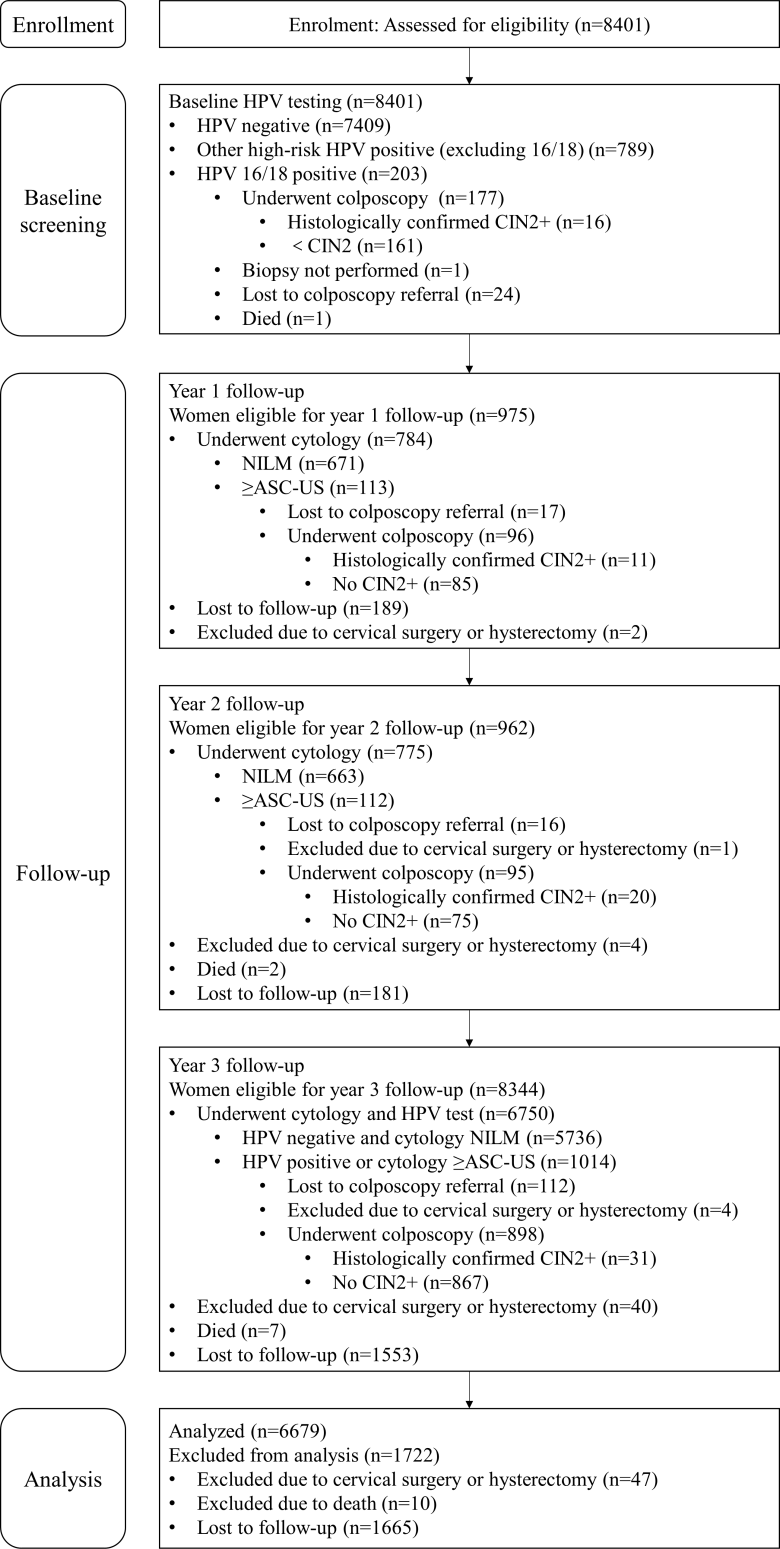
The flow diagram of participant enrollment, follow-up, and analysis. HPV, human papillomavirus; CIN2, cervical intraepithelial neoplasia grade 2; NILM, negative for intraepithelial lesion or malignancy; ASC-US, atypical squamous cells of undetermined significance.

The study was conducted in two phases, an initial baseline phase and a 3-year longitudinal follow-up phase, with the endpoint being a histopathological diagnosis of CIN2+ (Fig. 1). In the baseline phase, exfoliated cervical cells were collected from each eligible woman for HR-HPV testing. Women positive for HPV 16/18 were directly referred for colposcopy. Directed biopsies were taken from colposcopically abnormal areas; in unsatisfactory colposcopy (e.g., invisible squamocolumnar junction), random transformation-zone biopsies and endocervical curettage (ECC) were performed at the colposcopist’s discretion. Women with histologically confirmed CIN2+ were referred for clinical treatment and excluded from further follow-up.

At the first year and second year of follow-up, only women with positive HR-HPV at the baseline were recalled for cytology testing, and those with abnormal cytologic results (≥ASC US) were referred for colposcopy, with biopsy criteria as described above. At the third year of follow-up, all enrolled women except those diagnosed with CIN2+ at baseline and during the first 2 years of follow-up were recalled for cytology and HPV testing. Women with positive HR-HPV or abnormal cytology results were referred for colposcopy, following the same biopsy protocol. Women with negative HPV and cytology were not referred for colposcopy due to their very low expected CIN2+ risk, and histology was not ascertained in this group.

### HR-HPV testing

HR-HPV testing was performed using the HBRT-H14 assay (Hybribio, Chaozhou, China), which employs the commercially available HBRT-H14 kit (lot numbers: 20,170,301A, 20,171,001A, 20,180,901A, 20,200,502A, 20,200,603A, and 20,201,002A), following the manufacturer’s instructions. The assay uses TaqMan-based multiplex fluorescence PCR to target the E6 and E7 regions of 14 HR-HPV types (HPV 16, 18, 31, 33, 35, 39, 45, 51, 52, 56, 58, 59, 66, and 68), with concurrent separate genotyping of HPV 16 and HPV 18 from the pooled 12 other HR-HPV types. After denaturation of cervical exfoliated cell samples to release double-stranded HPV DNA, specific primers and fluorescently labeled oligonucleotide probes for HPV and the human β-globin gene (internal control) are used for real-time amplification and detection across four channels. Per the manufacturer’s instructions, samples were deemed HPV-positive if the cycle threshold (Ct) was ≤40 in the corresponding channel. The β-globin internal control was required to produce a valid signal (Ct ≤ 40) to confirm sample adequacy and amplification efficiency. Samples with initial invalid results were re-extracted and re-tested; no persistent invalid results remained after re-testing.

### Cytology and histology testing

The ThinPrep liquid-based cytologic test (TCT) was adopted to detect the cytology. All cytological samples were evaluated according to the Bethesda system with the following results: NILM, atypical squamous cell undetermined significance (ASC-US), atypical squamous cell—cannot exclude high-grade squamous intraepithelial lesion (ASC-H), low-grade squamous intraepithelial lesion (LSIL), high-grade squamous intraepithelial lesion (HSIL), squamous cervical carcinoma (SCC), and atypical glandular cells (AGC).

Biopsy samples obtained during colposcopy were processed and examined at each study center in Henan, Shanxi, and Guangdong Provinces. Each case underwent a double review by two pathologists, and the final diagnosis was made by the senior reviewer. p16 immunohistochemistry (with Ki-67 when needed) was used as an ancillary stain in ambiguous or suspected special-variant cases. The CIN grading reporting system was employed to document the diagnostic results, with the most severe histological findings determining the final diagnosis for each woman.

### Statistical analysis

The data were analyzed via SAS 9.4 and OpenEpi (available at http://www.openepi.com/). Continuous variables are expressed as the means and standard deviations (SDs), whereas categorical variables are presented as frequencies and percentages. The clinical endpoint for the study was defined as the detection of CIN2+ cases. In the analysis, “HPV 16/18 positive” refers to individuals who tested positive for HPV 16 and/or 18, regardless of coinfection with other HR-HPV types. “Other HR-HPV positive” includes only individuals who tested positive for HR-HPV types other than 16/18, excluding those with HPV 16/18 coinfection. The sensitivity, specificity, positive predictive value (PPV), and negative predictive value (NPV), along with their 95% confidence intervals (95% CIs), were calculated for the ability of HPV testing to detect CIN2+ and CIN3+ lesions at baseline and over a three-year cumulative period on the basis of initial HPV results. These diagnostic performance parameters were computed via the “Screening” module in OpenEpi, with the Wilson score method applied to derive the 95% CIs. The immediate and 3-year cumulative risks of developing CIN2+ and CIN3+ lesions (stratified by baseline HR-HPV status), together with their 95% CIs, were estimated via the “Two by Two Table” module in OpenEpi, with the Taylor series method used to determine the 95% CIs. Women who did not reach the endpoint event and failed to complete the third-year follow-up was considered lost to follow-up, and all analyses were performed on available data without imputation for missing values.

## RESULTS

### Characteristics of the study population

At baseline, 8,401 eligible women were enrolled in the study. During the 3-year follow-up, 57 (0.7%) women were excluded due to cervical surgery, hysterectomy, or death, and 1665 (19.8%) were lost to follow-up. Data from 6,679 qualified participants, with an average age of 45.6±8.2 years, were included in the final analysis. The overall HR-HPV positive rate was 11.4% (760/6,679), with HPV 16/18 positive rate of 2.3% (153/6,679) and other HR-HPV positive rate of 9.1% (607/6,679) (Table 1).

**TABLE 1:** Characteristics of the study population^*c*^

Characteristics	N	%
Age, years (mean ± SD)	45.6 ± 8.2	
30–39	1,776	26.6
40–49	2,707	40.5
50–64	2,196	32.9
Education		
Primary school and below	1,684	25.2
Middle school	3,192	47.8
High school and above	1,803	27.0
History of ever smoking		
Yes	1	0.0
No	6,678	100.0
History of ever alcohol drinking		
Yes	648	9.7
No	6,031	90.3
Baseline HPV infection		
HR-HPV positive	760	11.4
HPV 16/18 positive^*a*^	153	2.3
Other HR-HPV positive^*b*^	607	9.1
HPV negative	5,919	88.6

^*a*^ “HPV 16/18 positive” includes individuals positive for HPV 16 and/or 18, regardless of coinfection with other HR-HPV types.

^*b*^ “Other HR-HPV positive” includes individuals positive for HR-HPV types other than 16/18, excluding those coinfected with HPV 16/18.

^*c*^ SD, standard deviation; HPV, human papillomavirus; HR-HPV, high-risk HPV, including HPV types 16, 18, 31, 33, 35, 39, 45, 51, 52, 56, 58, 59, 66, and 68; other HR-HPV types include HPV types 31, 33, 35, 39, 45, 51, 52, 56, 58, 59, 66, and 68.

### Detection of CIN2/3+ lesions

**Fig 2:**
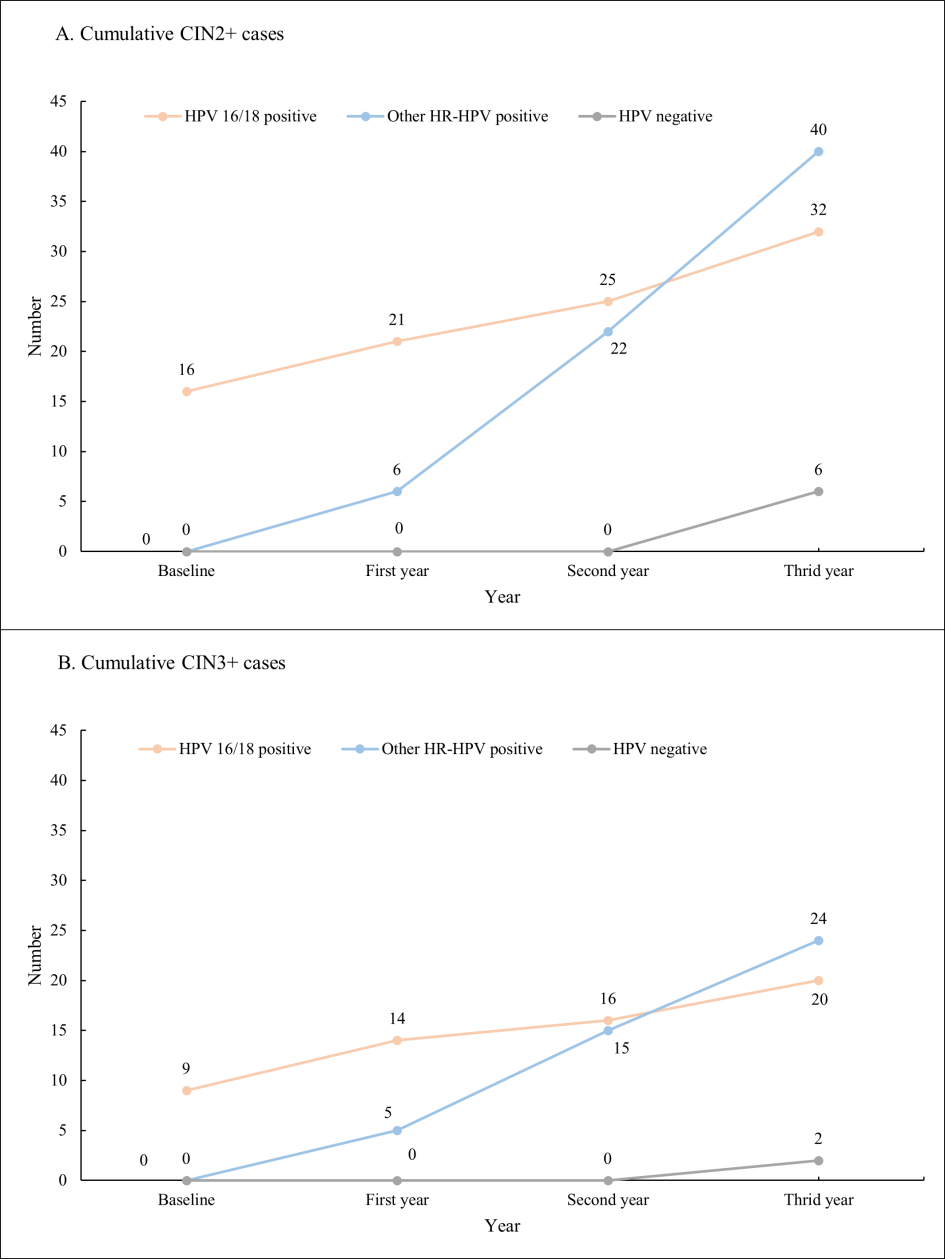
The cumulative number of CIN2+/CIN3+ cases by baseline HPV infection status. (**A**) Cumulative CIN2+ cases. (**B**) Cumulative CIN3+ cases. HPV, human papillomavirus; HR-HPV, high-risk HPV, including HPV types 16, 18, 31, 33, 35, 39, 45, 51, 52, 56, 58, 59, 66, and 68; CIN, cervical intraepithelial neoplasia.

We identified 16 CIN2+ cases at baseline, including 7 CIN2 cases and 9 CIN3+ cases. There were 11 cases diagnosed with CIN2+ in the first year, 20 in the second year, and 31 in the third year. Among the 78 CIN2+ cases, 32 were HPV 16/18 positive, 40 were other HR-HPV positive at baseline, and 6 were HR-HPV negative at baseline (Fig. 2).

### Clinical performance of HR-HPV testing in cervical cancer screening

During the baseline and cumulative 3 years, the overall sensitivity and specificity were 92.3% (95% CI: 84.2%–96.4%) and 89.6% (88.8%–90.3%) for HR-HPV positive against CIN2+ detection, and they were 41.0% (30.8%–52.1%) and 98.2% (97.8%-98.5%) for HPV 16/18 positive. For CIN3+ detection, the corresponding rates were 95.6% (85.5%–98.8%) and 89.4% (88.7%-90.1%) for HR-HPV positive, and 43.5% (30.2%–57.8%) and 98.1% (97.8%-98.4%) for HPV 16/18 positive (Table 2).

**TABLE 2:** Clinical performance of HPV testing during baseline screening and cumulative 3-year follow-up in cervical cancer screening^*c*^

Baseline HPV infection	Cases	Noncases	Sensitivity (%, 95% CI)	Specificity (%, 95% CI)	PPV (%, 95% CI)	NPV (%, 95% CI)
CIN2+						
HR-HPV						
Positive	72	688	92.3 (84.2–96.4)	89.6 (88.8–90.3)	9.47 (7.6–11.7)	99.9 (99.8–100.0)
Negative	6	5,913				
HPV 16/18						
Positive^*a*^	32	121	41.0 (30.8–52.1)	98.2 (97.8–98.5)	20.9 (15.2–28.0)	99.3 (99.1–99.5)
Negative	46	6,480				
Other HR-HPV						
Positive^*b*^	40	567	51.3 (40.4–62.1)	91.4 (90.7–92.1)	6.6 (4.9–8.9)	99.4 (99.1–99.5)
Negative	38	6,034				
CIN3+						
HR-HPV						
Positive	44	700	95.6 (85.5–98.8)	89.4 (88.7–90.1)	5.9 (4.4–7.9)	100.0 (99.9–100.0)
Negative	2	5,917				
HPV 16/18						
Positive^*a*^	20	124	43.5 (30.2–57.8)	98.1 (97.8–98.4)	13.9 (9.2–20.5)	99.6 (99.4–99.7)
Negative	26	6,493				
Other HR-HPV						
Positive^*b*^	24	576	52.2 (38.1–65.9)	91.3 (90.6–92.0)	4.0 (2.7–5.9)	99.6 (99.5–99.8)
Negative	22	6,041				

^*a*^ “HPV 16/18 positive” includes individuals positive for HPV 16 and/or 18, regardless of coinfection with other HR-HPV types.

^*b*^ “Other HR-HPV positive” includes individuals positive for HR-HPV types other than 16/18, excluding those coinfected with HPV 16/18.

^*c*^ HPV, human papillomavirus; PPV, positive predictive value; NPV, negative predictive value; CIN, cervical intraepithelial neoplasia; HR-HPV, high-risk HPV, including HPV types 16, 18, 31, 33, 35, 39, 45, 51, 52, 56, 58, 59, 66, and 68; other HR-HPV types include HPV types 31, 33, 35, 39, 45, 51, 52, 56, 58, 59, 66, and 68.

### Risk of CIN2/3+ by HPV infection status at baseline

The immediate risks of CIN2+ and CIN3+ for HPV 16/18 positive women were 11.2% (6.9%–17.5%) and 6.3% (3.2%–11.7%), respectively. Over the baseline and 3-year follow-up periods, the cumulative risk of CIN2+ was highest for HPV 16/18 positive individuals (20.9%, 15.2%–28.1%), followed by other HR-HPV-positive individuals (6.6%, 4.9%–8.9%) and HPV negative individuals (0.1%, 0.04%–0.2%). The cumulative absolute risk of CIN3+ was also highest in women who were HPV 16/18 positive (13.9%, 9.1%–20.6%), followed by the other HR-HPV-positive women (4.0%, 2.7%–5.9%) and the HPV-negative women (0.03%, 0.0007%–0.1%) (Table 3).

**TABLE 3:** Cumulative risk of CIN2+/3+ by baseline HPV infection status^*c*^

Baseline HPV infection	Cumulative absolute risk (%, 95 CI%)	Relative risk (95% CI)	
		HPV negative	Other HR-HPV+
CIN2+			
HR-HPV positive	9.5 (7.6–11.8)	93.5 (40.8–214.2)	–
HPV 16/18 positive^*a*^	20.9 (15.2–28.1)	206.3 (87.6–486.1)	3.2 (2.1-4.9)
Other HR-HPV positive^*b*^	6.6 (4.9–8.9)	65.0 (27.7–152.7)	–
HPV negative	0.1 (0.04–0.2)	–	–
Overall	1.2 (0.9–1.5)	–	–
CIN3+			
HR-HPV positive	5.9 (4.4–7.9)	175.0 (42.5–720.4)	–
HPV 16/18 positive^*a*^	13.9 (9.1–20.6)	411.0 (97.0–1742.0)	3.5 (2.0–6.1)
Other HR-HPV positive^*b*^	4.0 (2.7–5.9)	117.4 (28.1–499.6)	–
HPV negative	0.03 (0.00–0.13)	–	–
Overall	0.7 (0.5–0.9)	–	–

^*a*^ “HPV 16/18 positive” includes individuals positive for HPV 16 and/or 18, regardless of coinfection with other HR-HPV types.

^*b*^ “Other HR-HPV positive” includes individuals positive for HR-HPV types other than 16/18, excluding those coinfected with HPV 16/18; Cumulative risks are rounded to one decimal place except for values <0.1% (reported to two decimal places, with 95% CI lower limits <0.01 rounded to 0.00).

^*c*^ HPV, human papillomavirus; HR-HPV, high-risk HPV, including HPV types 16, 18, 31, 33, 35, 39, 45, 51, 52, 56, 58, 59, 66, and 68; other HR-HPV types include HPV types 31, 33, 35, 39, 45, 51, 52, 56, 58, 59, 66, and 68; CIN2, cervical intraepithelial neoplasia grade two; CIN3, cervical intraepithelial neoplasia grade three; “–” indicates data not applicable or not available.

## DISCUSSION

This study evaluated the clinical performance of the HBRT-H14 assay in detecting CIN2+ among women with NILM cytology in a multicenter prospective cohort in China. The assay demonstrated clear risk stratification: HPV 16/18 positive women had the highest risk, followed by other HR-HPV types, with HR-HPV negative women showing negligible risk. These gradients validate HBRT-H14 as an effective triage tool for identifying high-risk subgroups within NILM cytology.

The HR-HPV positivity in our NILM cohort (11.4%) exceeds the 7.5% reported from Shenzhen (20) and is higher than estimates from Italy (5.7%) (21) and Canada (6.9%) (22), yet remains within the reported range for Chinese general women (9.5%–22.2%) (23–27). Beyond regional exposure and healthcare access, methodological factors likely contribute to these differences: (i) variation in HPV assays and cut-offs, (ii) interlaboratory and interobserver variability in cytology thresholds that influences the NILM denominator, and (iii) known discordance among HPV assays, particularly in cytology-normal populations (28–30). Taken together, these factors can elevate apparent HPV prevalence in NILM cohorts without necessarily indicating missed diagnoses. Future cross-study comparisons should specify the HPV assay, analytical cut-offs, and cytology quality metrics to contextualize prevalence estimates.

HPV testing in this study showed high sensitivity and good specificity for detecting CIN2+, supporting its use in contemporary screening pathways (31–33). Cytology, while traditional, has lower sensitivity and greater subjectivity (13, 34), underscoring its role mainly as triage after a positive HPV test. Genotyping adds clinical value by directly identifying oncogenic types (notably 16 and 18). Evidence indicates that risk varies by genotype and with infection persistence (34, 35). In particular, same-genotype persistence confers a substantially higher risk of high-grade CIN than genotype switch or transient infection (35), highlighting the clinical importance of genotype-specific monitoring for refined risk stratification and timely intervention. This approach helps identify women who warrant closer follow-up, improving early detection while minimizing unnecessary procedures.

Our study highlights the risk stratification potential of HPV 16/18 genotyping. Women positive for HPV 16/18 had the highest 3-year CIN2+ risk (20.9%) and immediate CIN3+ risk (6.3%), exceeding the 4% threshold for direct colposcopy recommended by the 2019 American Society for Colposcopy and Cervical Pathology (ASCCP) guidelines (36). In contrast, women positive for other HR-HPV types had a 3-year cumulative CIN3+ risk of 4%, exceeding the 0.55% threshold for 1-year follow-up, and warranting short-interval surveillance. The elevated risk observed in HPV 16/18-positive women is consistent across domestic studies (20, 23, 32) and international cohorts, including the ATHENA trial (37, 38), as well as studies from Norway (39), Canada (40), and Italy (41). This global alignment confirms the value of genotyping in risk-adapted management. Consistent with this evidence, several national guidelines, including those in the United States and Canada (36, 42), recommend that HPV 16/18-positive women be referred directly for colposcopy. This strategy streamlines triage, reduces diagnostic delays, and optimizes clinical resource allocation.

It’s worth noting that among the 14 HR-HPV infections in this study, HPV 16/18 accounted for 20.1% (153/760), while the other 12 HR-HPV types comprised 79.9% (607/760). Although HPV 16/18 are the primary drivers of cervical cancer (20), other HR-HPV types showed substantial prevalence and contributed to a notable proportion of CIN2+ lesions. Recent studies found that risks vary among these types, such as HPV 31 and HPV 33 (23, 43), highlighting the clinical relevance of extended genotyping. Furthermore, with HPV vaccination expected to reduce HPV 16/18 prevalence, the relative burden of other HR-HPV types may increase. Extended genotyping has been incorporated into several national screening programs (44–47), but further evaluation is needed to optimize its use and interpretation. These considerations underscore the need to adapt future cervical cancer screening strategies, including risk-based triage and population-specific implementation of extended genotyping, in the context of increasing HPV vaccination coverage.

While the study provides valuable insights, it has several limitations. First, there was a 19.8% loss to follow-up, which may have affected the risk estimates; however, baseline characteristics did not differ significantly between women who completed follow-up and those lost, suggesting minimal bias. Second, women negative for both HPV and cytology were not referred for colposcopy, which could introduce verification bias and potentially miss a small number of CIN2+ lesions; nevertheless, adjudication data from the ATHENA trial indicate that CIN2+ is very rare in double-negative women (48). Third, as the cohort primarily comprises Chinese women, differences in healthcare infrastructure, environmental factors, and population genetics across other regions may influence the generalizability of these findings.

In conclusion, in this national multicenter prospective cohort, the HBRT-H14 assay showed strong clinical performance for detecting CIN2+, and HPV 16/18 genotyping provided meaningful risk stratification among women with NILM cytology. These findings support integrating HBRT-H14 into HPV-based screening pathways with HPV 16/18 genotyping and cytology triage of other types (not 16/18).

## Data Availability

The data sets used and/or analyzed during the current study are available from the corresponding author on reasonable request.
